# Impact of blue-light filtering intraocular lens implantation on the quality of sleep in patients after cataract surgery

**DOI:** 10.1097/MD.0000000000005648

**Published:** 2016-12-23

**Authors:** Xue Feng, Ke Xu, Yansheng Hao, Hong Qi

**Affiliations:** a Department of Ophthalmology, Peking University Third Hospital, Beijing; b Key Laboratory of Vision Loss and Restoration, Ministry of Education, China; c Moslem Hospital, Beijing, China.

**Keywords:** blue-light filtering intraocular lens, cataract, circadian rhythm, quality of sleep

## Abstract

**Background:**

There are 2 main types of intraocular lens (IOL) currently implanted in patients after cataract surgery. Till now, we do not know exactly what the effect of intraocular lens implantation will be on the quality of sleep after cataract surgery.

**Methods:**

The binocular BF-IOL (BF-IOL Groups) and UVB-IOL (UVB-IOL Groups) implantations were performed in 60 and 59 cataract patients, respectively. Pittsburgh Sleep Quality Index (PSQI) questionnaires were administered to evaluate the quality of sleep in patients preoperatively, 1 month (1 m) and 12 months (12 m) postoperatively. Independent sample test and χ^2^ test were used to evaluate the difference between the 2 groups; one-way ANOVA was used to evaluate the difference preoperatively and postoperatively in each group. The rank sum test was used for statistical analysis of 7 independent sleep components in PSQI.

**Results:**

As compared to preoperatively, the PSQI overall scores in both groups improved significantly postoperatively (*P* = 0.00 at 1 m and 12 m). Among the 7 components of PSQI, 2 of them (sleep latency and daytime dysfunction) improved greatly postoperatively in both groups (*P*< 0.05). Although the improvement of PSQI overall score in the UVB-IOL group was greater than that in the BF-IOL Group only at early time (1 m) postoperatively (*P* = 0.00), but not late time (12 m, *P* > 0.05) after the cataract surgery.

**Conclusion:**

The sleep quality of cataract patients improved after IOL implantation, regardless of the type of IOL, suggesting that BF-IOL might serve as an alternative to conventional UVB-IOL without a detrimental effect on quality of sleep after cataract surgery.

## 1 Introduction

The retinal pigment epithelium (RPE) cells could be damaged by the radiation of visible light of short wavelength^[1]^ (between 400 and 500 nm). This retinal damage is in accordance with the pathophysiology of age-related macular degeneration (AMD). There are 2 main types of intraocular lens (IOL) currently implanted in patients after cataract surgery, which differ in their transmission properties: conventional ultraviolet blocking (UVB)-IOL and blue-light filtering (BF)-IOL. The BF-IOL, which is able to block ultraviolet rays as well as purple and blue visible light between 400 and 500 nm, was designed with the purpose of retinal protection.

Human circadian rhythm is mediated by melatonin secretion, which is regulated through the visual signal from the retina to the pineal gland.^[2]^ The dim light at night promotes melatonin secretion which keeps people sleep, whereas the high intensity of light at daytime, especially blue light inhibits melatonin secretion which keeps people awake. Since blue light have maximum inhibitory effect on melatonin secretion,^[3–5]^ implying that BF-IOL implantation after surgery might influence the circadian rhythm of cataract patient and further have adverse effects on the quality of sleep.^[6–9]^ Although other investigators presented their different opinions, Landers et al^[10]^ reported that BF-IOL implantation had no adverse effect on the sleep quality in cataract patients after the surgery as comparing with the conventional UVB-IOL implantation, but their study showed the limitation with short of preoperative data. Wei's study^[11]^ indicated that BF-IOL could improve the quality of sleep after cataract surgery with 2 months follow-up, but their study was short of control group. Till now, we do not know exactly what the long-term effect of BF-IOL implantation will be on the quality of sleep after cataract surgery. In this prospective cohort study, we used the Pittsburgh Sleep Quality Index to compare the effect of BF-IOL implantation to UVB-IOL with 12 months follow-up to evaluate the quality of sleep in patients before and after cataract surgery.

## 2 Material and methods

### 2.1 Patients

In total, 152 patients who had bilateral cataract surgery in the Department of Ophthalmology, Peking University Third Hospital during the 12-months period between January and December, 2013, were recruited in this study. They were divided into 2 groups: BF-IOL group or UVB-IOL group. The binocular BF-IOLs (Acrysof IQ, Alcon Laboratories) and conventional UVB-IOLs (Tecnis, Abbott Medical Optics, Inc) were implanted in 77 and 75 cataract patients, respectively. All the included cataract patients had lens opacity≥N_2_ before the surgery according to the Lens Opacity Classification System II (LOCS II). LOCS II is a grading system for cataract according to the transparency of the lens. Focusing on the nucleus hardness, it can be divided into 4 levels (level N_0_: transparent nucleus, colorless; N_1_: soft nucleus, yellow-white color; N_2_: medium hardness nucleus, yellow color; N_3_: stiff nucleus, dark brown color). The target refraction for IOL implantation is emmetropia in all cases. All the operations were performed by the same skilled doctor.

Exclusion criteria included patients who were inability to complete a questionnaire due to confusion or dementia, suffered from high myopia and retinal optic neuropathy with light absorption problems (including pigmentary degeneration or ischemic optic neuropathy), with color vision problems (diabetes, glaucoma) or color blindness. Individuals with sleep disorders; treatment with benzodiazepines; with a diagnosis of physical or psychiatric conditions, with a history of head injury, past alcohol or drug abuse were also excluded from the study. The study was performed after ethics committee of Peking University Third Hospital gave ethical approval. Informed consent forms were signed by all patients who participating in the study.

### 2.2 Questionnaire interviews

The questionnaire administered in this study was Pittsburgh Sleep Quality Index (PSQI) (Table 1).^[12]^ It is a self-rating sleep questionnaire. It has been used in clinic for almost 20 years and over that time has consistently shown robust validity and reliability^[13–15]^ to evaluate the quality of sleep by scoring 7 different independent sleep components. The PSQI consisted of 17 self-rated questions corresponding to 7 components which included subjective sleep quality, sleep latency, sleep duration, habitual sleep efficiency, sleep disturbances, use of sleeping medication, and daytime dysfunction. Each component was graded from 0 to 3, and the accumulated scores were measured as overall score, ranging from 0 to 21. A higher score correlated with poorer sleep quality, and scores ≥6 indicated poor quality of sleep. It usually took 5 to 10 minutes for patients to complete the questionnaire. In this study, the PSQI questionnaire interview was conducted for each participant preoperatively, at 1 m and 12 m postoperatively.

**Table 1:**
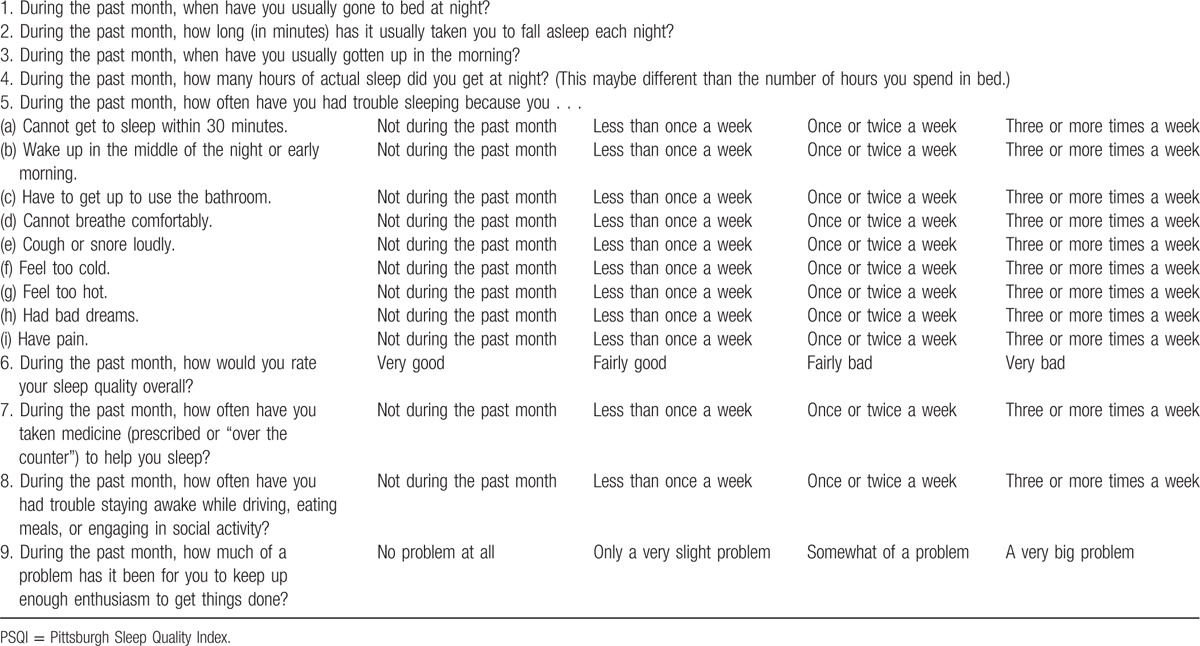
Pittsburgh Sleep Quality Index (PSQI) questionnaire^[12]^.

### 2.3 Statistics

Statistical Analysis System softwar (SPSS19.0 software) was used in this study. The independent sample test and χ^2^ test were used to evaluate the difference between the 2 groups (BF-IOL group and UVB-IOL group). One-Way ANOVA was used to evaluate the difference of PSQI overall scores among the 3 time points (preoperatively, 1 m and 12 m postoperatively) in each group. The data of 7 components in PSQI was not normal distributed, so the rank sum test was used for statistical analysis. A *P* value of < 0.05 was considered significant.

## 3 Results

Of the 152 recruited patients, 33 patients were lost during the 12-months follow-up period because of the changed telephone number or address. There were 60 in the BF-IOL group (32 men, 53%; 28 women, 47%), and 59 (30 men, 51%; 29 women, 49%) in the UVB -IOL group who finished all the PSQI Questionnaire interviews enrolled in this study. The mean age of patients in 2 groups (BF-IOL group and UVB-IOL group) was 74 ± 5.70 years and 75 ± 5.50 years, respectively. There was no significant difference shown between the 2 groups (*t* = 0.41, *P* = 0.30). There was also no significant difference shown in the ratio of sex between the 2 groups (χ^2^ = 0.01, *P* = 0.75). There were 40 (66.7%) patients in the BF-IOL group with preoperative PSQI overall score≥6 and 40 (67.8%) patients in the UVB-IOL group with preoperative PSQI overall score≥6. There was no significant difference shown in the ratio of poor sleep between the 2 groups (χ^2^ = 0.02, *P* = 0.90). The statistics of preoperative data in the 2 groups were shown in Table 2.

**Table 2:**
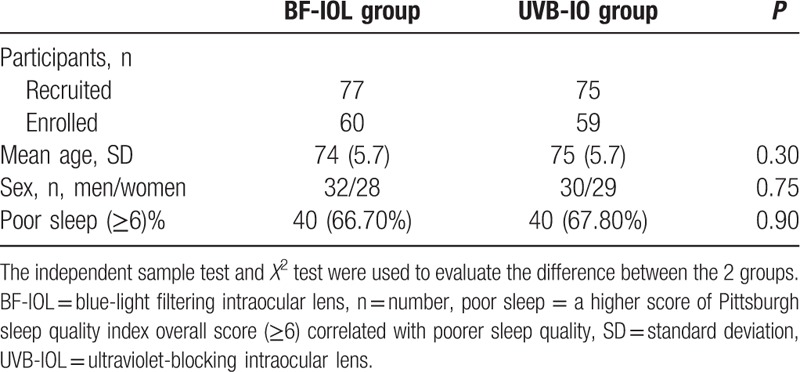
The Statistics of Descriptive Data before the cataract surgery in the two groups.

**Figure 1:**
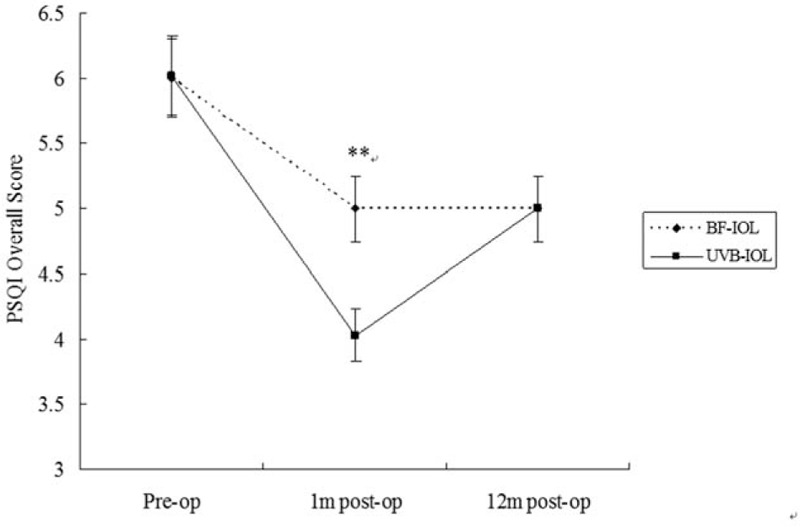
PSQI overall scores in the BF-IOL group (dotted line) and UVB-IOL group (solid line) at 3 time points (pre-op, 1 m post-op, 12 m post-op). The independent sample test was used to evaluate the difference of PSQI overall scores between the 2 groups at 3 time points. ^∗∗^, *P* < 0.01. 1 m post-op = 1 month after the surgery, 12 m post-op = 12 months after the surgery, BF-IOL = blue-light filtering intraocular lens, Pre-op = before the surgery, PSQI = Pittsburgh Sleep Quality Index, UVB-IOL = ultraviolet-blocking intraocular lens.

As Table 3 and Fig. 1 shown, PSQI overall scores in the BF-IOL group was 6.00 ± 1.09, preoperatively, which go down to 5.00 ± 1.09 at 1 m postoperatively and that was 5.00 ± 1.04 at 12 m postoperatively. There were significant differences of PSQI overall scores among the 3 time points (preoperatively, 1 m and 12 m postoperatively) in the BF-IOL group (*F*=17.35,*P* = 0.02). The PSQI overall score in patients who underwent BF-IOL implantation improved significantly at 1 m postoperatively compared to that preoperatively (*P* = 0.00). This effect of improvement sustained till 12 m postoperatively as compared to the results preoperatively (*P* = 0.00). PSQI overall scores in the UVB-IOL group was 6.02 ± 1.04 preoperatively, which go down to 4.02 ± 1.09 at 1 m postoperatively and that was 5.00 ± 1.09 at 12 m postoperatively (Table 3). There were significant differences of PSQI overall scores among the 3 time points (preoperatively, 1 m and 12 m postoperatively) in the UVB-IOL group (*F* = 43.68,*P* = 0.00). The PSQI overall score of patients who underwent UVB-IOL implantation improved significantly at 1 m postoperatively compared to that preoperatively (*P* = 0.00). This effect of improvement also sustained till 12 m postoperatively as compared to the results preoperatively (*P* = 0.00). There was no significant difference in PSQI overall scores between the 2 groups preoperatively (*t* = 0.09, *P* = 0.93) and at 12 m postoperatively (*t*= 0.08, *P* = 0.93), whereas the scores were significantly higher in the BF-IOL group at 1 m postoperatively than that in the UVB-IOL group (*t* = 4.92, *P* = 0.00, Fig. 1).

**Table 3:**
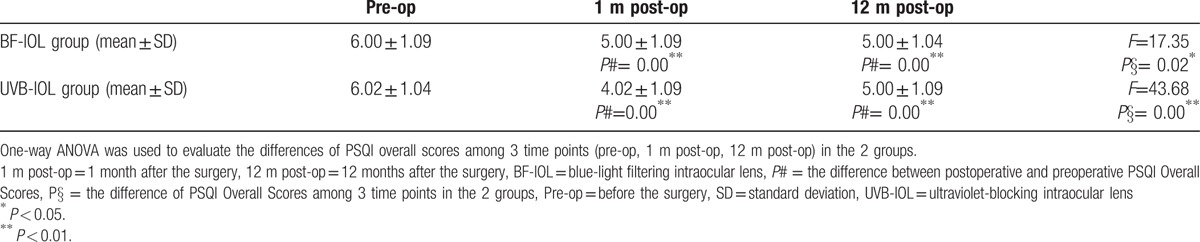
PSQI overall scores before and after cataract surgery in the 2 groups.

As Table 4 shown, the median score of sleep latency in the BF-IOL group decreased significantly at 1 m (*Z*=12.30, *P* = 0.00) and 12 m (*Z*=12.01, *P* = 0.00) postoperatively, which also happened in the UVB-IOL group postoperatively (*Z*=23.50, *P* = 0.00 at 1 m and *Z*=11.35, *P* = 0.00 at 12 m). The median score of daytime dysfunction in the BF-IOL Group decreased significantly at 1 m (*Z*=6.59, *P* = 0.01) and 12 m (*Z*= 6.59, *P* = 0.01) postoperatively, which also happened in the UVB-IOL group postoperatively (*Z*=14.20, *P* = 0.00 at 1 m and *Z*=8.44, *P* = 0.00 at 12 m). There were no significant differences shown in other 5 independent sleep components (subjective sleep quality, sleep duration, habitual sleep efficiency, sleep disturbances, and use of sleeping medication) among the 3 time points (preoperatively, 1 m and 12 m postoperatively) in each group. There were also no significant differences between the 2 groups for each 7 independent sleep component before and after cataract surgery (all *P *> 0.05).

**Table 4:**
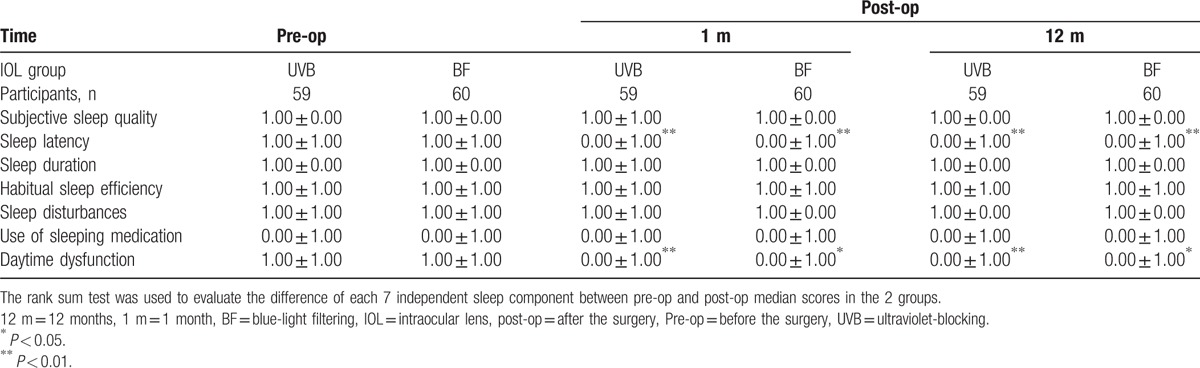
Median Scores of the PSQI before and after cataract surgery in the 2 groups.

## 4 Discussion

BF-IOL was designed with the purpose of retinal protection. Then, how the BF-IOL implantation will affect the quality of sleep in the cataract patient after surgery with comparing to their preoperative quality of sleep? In our study, we found that there were significant differences shown in PSQI overall scores among the 3 time points (preoperatively, 1 m and 12 m postoperatively) in the BF-IOL group (*P *< 0.05). The quality of sleep in patients who underwent BF-IOL implantation improved at 1 m postoperatively as compared to that preoperatively (*P *< 0.01). Our result was consistent with Wei's study.^[11]^ Although this effect of improvement would sustain till 12 m postoperatively (*P *< 0.01), among the 7 independent sleep components, 2 of them (sleep latency and daytime dysfunction) improved greatly at both 1 m and 12 m postoperatively (all *P *< 0.05). There would be 2 reasons for the improvement on the quality of sleep in the BF-IOL group after the cataract surgery. Once the cataract has been removed, the overall light transmission increased. Although the BF-IOL is able to block part of purple and blue visible light between 400 and 500 nm, the residual blue light transmission of BF-IOL is sufficient to suppress the melatonin at daytime. The circadian rhythm regulatory system was responded to the sudden increased light exposure.^[16]^ The inhibition of melatonin secretion, which keeps people awake at daytime, further improves the quality of sleep at nighttime. Another reason for the improvement of sleep quality was the recovery of visual acuity after the cataract surgery. The increased visual acuity making peoples do more physical exercise at daytime, so they would feel sleepier at nighttime.

Secondly, how the BF-IOL implantation in cataract patient will affect the quality of sleep after the surgery with comparing to the conventional UVB-IOL implantation? As Fig. 1 shown in our study, there was no significant difference in the preoperative PSQI overall score between the 2 groups (*P *> 0.05). However, the improvement of sleep quality in the UVB-IOL group was greater than that in the BF-IOL group only at early time (1 m) postoperatively (*P *< 0.01), but not late time (12 m, *P *> 0.05). Moreover, there were no significant differences shown between the 2 groups in each 7 independent sleep component at any 3 time points: preoperatively, 1 m and 12 m postoperatively (all *P *> 0.05). The greater of improvement in the UVB-IOL group on the quality of sleep than that in the BF-IOL group is because that the blue light transmittance of the BF-IOL is lower than that of UVB-IOL at early time. As time goes by, patients adapted to the new conditions. So there was no significant difference shown on the quality of sleep between the 2 groups at late time postoperatively. Our conclusion is consistent with Landers’ finding^[10]^ that BF-IOL implantation had no adverse effect on the sleep quality in cataract patients after the surgery as comparing with the conventional UVB-IOL implantation.

In summary, the sleep quality of cataract patients improved after IOL implantation, regardless of the type of IOL, suggesting that BF-IOL might serve as an alternative to conventional UVB-IOL without a detrimental effect on quality of sleep after cataract surgery.
